# ComOn Coaching: Study protocol of a randomized controlled trial to assess the effect of a varied number of coaching sessions on transfer into clinical practice following communication skills training

**DOI:** 10.1186/s12885-015-1454-z

**Published:** 2015-07-07

**Authors:** Marcelo Niglio de Figueiredo, Bärbel Rodolph, Carma L Bylund, Tanja Goelz, Pia Heußner, Heribert Sattel, Kurt Fritzsche, Alexander Wuensch

**Affiliations:** 1 Department of Psychosomatic Medicine and Psychotherapy, Freiburg University Medical Center, Hauptstr. 8, D-79104, Freiburg, Germany; 2 Clinic of Dermatology and Venereology, Freiburg University Medical Center, Hauptstr. 7, D-79104, Freiburg, Germany; 3 Department of Psychosomatic Medicine and Psychotherapy, Klinikum rechts der Isar, Technical University Munich, Langerstr. 3, D-81675, München, Germany; 4 Department of Medical Education, Hamad Medical Corporation; Doha-Qatar, Weill-Cornell Medical College – Qatar, Doha, Qatar; 5 Center for Pediatrics, Freiburg University Medical Center, Mathildenstr. 6, D-79106, Freiburg, Germany; 6 Department of Haematology and Internal Oncology, Interdisciplinary Psycho-Oncology Center, University Clinic of Munich – Grosshadern, Marchioninistr. 15, D-81377, München, Germany

**Keywords:** Communication skills training, Transfer, Coaching

## Abstract

**Background:**

Communication skills training has proven to be an effective means to enhance communication of health care professionals in oncology. These effects are well studied in standardized settings. The question of transferring these skills into clinical consultations remains open. We build up on a previous developed training concept consisting of a workshop and coaching. This training achieved a medium effect size in two studies with standardized patients. In the current study, we expanded and manualized the coaching concept, and we will evaluate effects of a varied number of coaching sessions on real clinical consultations. Our aim is to determine how much coaching oncologists need to transfer communication skills into clinical practice.

**Methods/design:**

Physicians of two German medical centers will participate in a workshop for communication skills and will be randomized to either a group with one coaching session or a group with four coaching sessions following the workshop. The participation is voluntary and the physicians will receive medical education points. Consultations held by the participating physicians with actual patients who gave their informed consent will be filmed at three time points. These consultations will be evaluated by blinded raters using a checklist based on the training content (primary outcome). Secondary outcomes will be the self-evaluated communication competence by physicians and an evaluation of the consultations by both physicians and patients.

**Discussion:**

We will evaluate our communication training concept on three levels – rater, physician and patient – and concentrate on the transfer of communication skills into real life situations.

As we emphasize the external validity in this study design, limitations will be expected due to heterogeneity of data. With this study we aim to gain data on how to improve communication skills training that will result in better patient outcomes.

**Trial registration:**

German Clinical Trials Register DRKS00004385.

**Electronic supplementary material:**

The online version of this article (doi:10.1186/s12885-015-1454-z) contains supplementary material, which is available to authorized users.

## Background

Good communication is one key to good cancer care [1, 2]. Earlier studies have described positive effects of effective physician-patient communication (PPC) for clinicians (greater job satisfaction, better time management, lower burnout level), for patients (higher satisfaction, greater adherence to treatment, reduced anxiety, increased recall of information and improved understanding) as well as for the treatment as a whole (better patient health outcomes) [3]. These results triggered several programs and research for PPC in the last 20 years. In order to improve PPC in oncology, communication skills training (CST) plays a critical role, which is reflected in the increasing number of solid studies on CST for health care professionals (HCP). While a 2004 review lists only three high quality studies before 2001 [4], a newer Cochrane review found 15 eligible studies until 2012 [5]. This last review reports, in agreement with previous studies [6, 7] that CST shows small to medium effect sizes (ES) while their long term effects tend to be small.

While empirical evidence shows that communication skills can be taught successfully, the question remains how to teach them effectively. Earlier literature suggests that too short CSTs are ineffective [8, 9] and a European consensus meeting proposed in 2009 that “a course of at least 3 days appears necessary to ensure transfer into clinical practice”. They emphasize that “supervision and periodic booster sessions are a promising add-on” [10]. Furthermore the Cochrane Review [5] reports that CSTs tend to show higher ES when they are assessed with actor-patients than with real patients. These findings corroborate earlier studies [11, 12] and imply that transfer from workshop into clinical everyday life does not necessarily happen automatically. In fact Heaven et al. [13], investigating the effect of supervision on transfer after a CST for nurses, observed that only the group under supervision showed some signs of transfer into the clinical praxis after three months. The results of this study were not statistically significant and authors discussed that, among other methodological problems, the characteristics of the supervision may have had great impact on the outcome, as discussed below.

Several training concepts have been presented in order to facilitate the transfer into the clinical practice integrating coaching, supervision and booster sessions after CST. After a 2-days workshop using the Swiss-Model, Stiefel et al. (2010) [14] offered the trained HCP 4–6 individual supervisions and completed the training program with a booster 0,5-day workshop six months after the first workshop. Razavi et al. (2003) [15] offered six consolidation workshops (3 hours per evening) during the six months after a 19-hour basic training (2 days (8 hours each) and one evening (3 hours)). In the study by Heaven et al. (2006) [13] 4 weekly supervisions of 3 h followed a 3 day long workshop while the training from Butow et al (2008) [16] consisted of a 1.5-day workshop followed by four 1.5-hour video-conferences in monthly intervals. The video-conferences proposed by Butow et al. (2008) [16] allow physicians in regions difficult to access to participate in a long term training and Langewitz et al. (2010) [17] added more flexibility to the Swiss-model offering the supervision per telephone. This last training was accompanied of didactic materials such as a training DVD and information booklets. Liénard et al. (2010) [18] proposed a 40-hour training program consisting of 30 h CST and 10 h stress management spread bimonthly over 8 months. Curtis et al. (2013) [19] offered eight four hour training-blocks, each one consisting of a brief didactic overview with a demonstration role-play by facilitators, skills practice using simulation (simulated patients, family, or clinicians); and reflective discussions. All these approaches have in common that (1) a single workshop is replaced or complemented by shorter interventions spread over a longer time, so that (2) the HCP can practice and reflect over what they have learned and (3) creative solutions have been found for feasibility problems.

Whereas these programs show clear strengths, their limitations have to be faced as well. Although they all use coaching/supervision and/or booster sessions, few studies have researched these elements systematically. While in most studies with standardized patients presented by the trainers, learning goals are set by the training program, Fallowfield and Jenkins (2004) [20, 21] stressed the importance of self-defined goals for the motivation and success of the training. The form of the feedback also seems to play a very important role and Heaven et al. (2006) [13] discuss that video recorded real consultations would be a better basis for supervision. Furthermore, the general emphasis on the cognitive and behavioral dimension and modeling is at the cost of intrapsychic aspects such as the physicians’ emotions and affective reactions. These considerations seem to indicate that CST (1) should be adapted to the individual needs of the HCP, (2) should be based on real conversations, (3) should offer time for in-depth reflection of the consultation, and (4) video recorded conversations should be used for the supervision.

The CST in this study builds up on a previous CST, developed by our research group for oncologists. It consisted of a 1½-day (12 h) workshop followed by an individual coaching appointment (30 min). It achieved medium ES in short-term measurements [22, 23]. These two studies were limited in their external validity as they were thematically restricted (transition from curative to palliative care and discussion of clinical trials, respectively) and as the assessments were done with standardized patients. In order to reduce these limitations a new study was conceptualized, where a broader spectrum of oncological consultation with real patients is considered. Moreover, our experience of the conducted studies suggests that the coaching represents an important addition to the workshop, so that the coaching concept was expanded, manualized and now systematically evaluated. The training is individualized through a focus on individual learning goals, individual role play scenarios and integrating video recorded real consultations of the participants.

### Objectives

The main purpose of this project is to determine the increase on efficacy of the ComOn CST when followed by intensive coaching. Primary outcome is the blinded rating of real physician-patient consultation by means of a checklist covering relevant communication skills. Secondary outcomes are the self-evaluation by the physicians and the evaluation by the patients. As an explorative objective, we will assess on how communication skills are improved by focusing on individual learning goals.

### Hypotheses

#### Primary outcome

Physicians of the Intervention group (IG) with four coaching sessions show a greater increase in communication skills after the training than physicians of the Control group CG with one coaching session, as assessed by blinded raters applying a checklist.

#### Secondary outcomes

Physicians of the IG rate themselves better after four coaching sessions regarding their communication skills than physicians of the CG assessed by a questionnaire.

Patients appraise the communication skills of the physicians of the IG better than the ones of the CG assessed by a questionnaire.

Physicians of the IG appraise themselves better than physicians of the CG three months after the intervention.

#### Exploratory outcome

Physicians of the intervention group improve in their individual learning goals more than physicians of the control group. They will pick up the need of patients better than the control group.

## Methods/design

In a bicentric trial, physicians will be recruited in medical centers of Freiburg and Munich, Germany, to participate in a CST. Real physician-patient consultations are video recorded and rated by independent, trained raters and evaluation questionnaires are filled out by both physicians and patients. The treatment integrity is ensured through regular supervision of the trainers. Randomization is done by an independent statistician.

### Study design

**Fig. 1:**
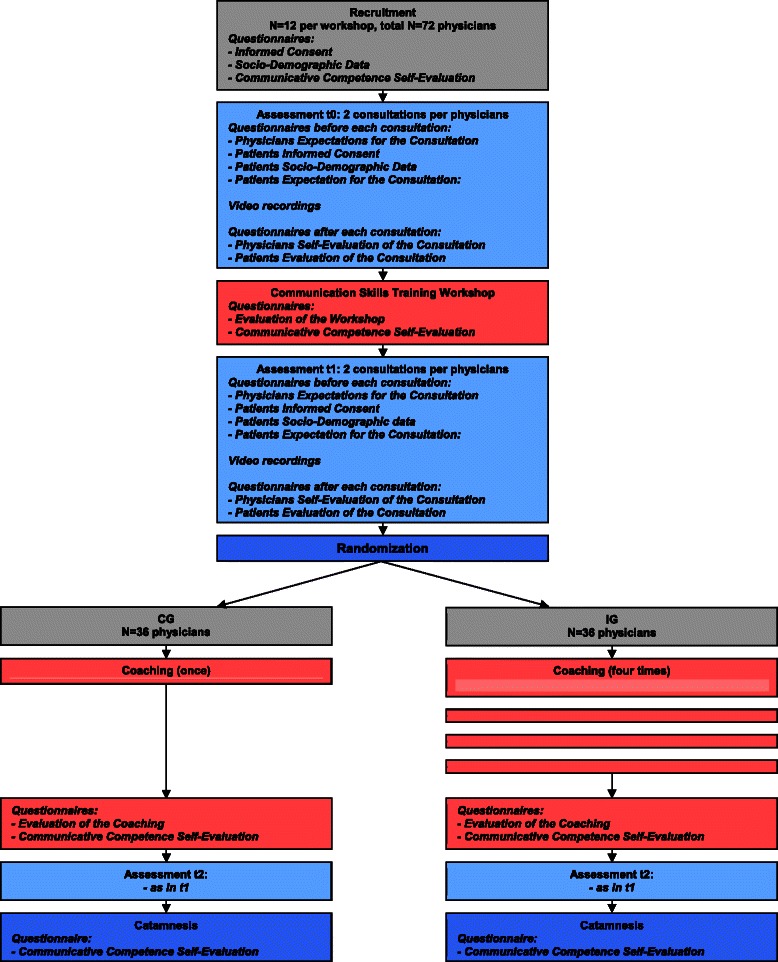
Study design

It is a randomized controlled trial. The Study design is shown in Fig. 1.

### Participants

Physicians of the University Medical Center Freiburg, Germany, and the Medical Center of the University Hospital *Klinikum rechts der Isar*, Munich, Germany, as well as affiliated hospitals are invited for a CST. 28 continuing medical education points (CME) for participation and a certificate for a psychosomatic basis care qualification necessary for some medical specialties can be achieved. Prerequisites for participation are involvement in cancer care, written informed consent for the study and completing procedures before coaching sessions, i.e. assessment t0 and t1 as well as the workshop. The training is open to physicians of any field of oncology and setting, i.e. in or outpatients.

### Trainer

All trainers have field competency in oncology as psycho-oncologists and are experienced in conducting communication skills training for physicians. All trainers were trained for the communication workshops as well as for the coaching sessions by the principal investigator AW. Training integrity will be achieved by supervision of the trainers.

### Training

The training combines a 1.5 day workshop on communication skills and an in depth one-to-one coaching after the workshop. All participants will take part in the workshop first. After that two groups will be randomized: The intervention group (IG) with four coaching sessions and the control group (CG) with only one coaching.

#### Workshop

The workshop will last 1.5 days and is 12.75 h long. It is designed for up to 12 physicians, divided up in small groups of three or four physicians and covers three modules: (1) Introduction, (2) Video-analysis and practicing communication skills in role play with standardized patients and (3) a final feedback round.

*First Module*: In the beginning of the workshop and after a warming up, a scene of the film “Stopped on Track” [2011] is shown. The scene shows a neurosurgeon disclosing diagnosis of an inoperable glioblastoma to a patient and his wife. The scene is used to trigger discussion about good communication. After a short communication exercise theoretical background about the need and impact of good communication is presented and a presentation of the SPIKES-protocol [24] in the form of the checklist items finishes the theoretical input.

The most important communication skills, which also constitute the checklist items, are summarized and handed to participants in the form of a pocket-sized ‘Memory Card’. In the end of this module the physicians are taught rules to provide specific and constructive feedback for the Second Module.

*Second Module*: The group is divided into small groups of three to four participants. First, the physicians formulate learning goals by watching videos of their own consultations with cancer patients, which were recorded before the workshop. Participating colleagues give feedback and the trainer establishes a link to the theoretical information. Learning goals are then trained in individual situations: Each physician informs the actor-patients about a current clinical situation in their personal clinical life in order to develop a role play scenario. The clinical situation is first played the way the physician had experienced it in the real situation, afterwards actor-patients, colleagues and the trainer give their feedback and make suggestions as to how to improve communication skills. The suggestions are tried out and skills are practiced again in the subsequent role play situation. This procedure is repeated on the next day, so each participant has the chance to practice two different role play scenarios. They are invited to practice two different situations: one, how to structure information, and the other one, how to handle emotional topics.

*Third Module*: In the end of the workshop everyone participates in a feedback round and is asked to formulate a take home message for future practice. The workshop is finished by an evaluation questionnaire.

#### Coaching

##### Theoretical background and pre-analysis

**Fig. 2:**
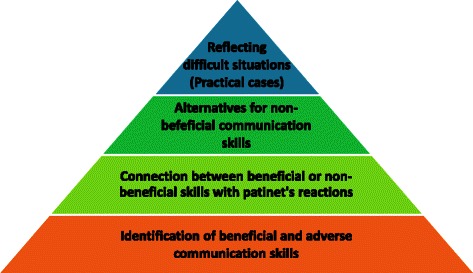
Learning pyramid

The coaching sessions build up on experiences in former projects and the authors’ cognitive-behavioral psychotherapy background. A modified learning pyramid (see Fig. 2) based on the Miller Pyramid [25] and the self-regulation model for behavior change [26] constitutes the theoretical background. In the first step participants learn to recognize beneficial and adverse communicative techniques and to identify them in their own behavior. The second step aims at becoming aware of the connection between one’s own (beneficial or adverse) communicative behavior and the (likewise beneficial or adverse) reaction of the interlocutor and vice versa. As soon as this connection has been elaborated, alternative behavior and techniques are gathered which potentially lead to more beneficial interactions (third step). These alternatives are tried out and reflected in consequence. The last step includes reflecting difficult situations in practice. It is discussed how the new beneficial techniques can be consolidated and adjusted to the everyday needs of the participants.

Based on the individual goals defined in the workshop and on the checklist, the trainers watch the two videos and evaluate them before the first appointment with regard to the questions “What was good?”/“What could have been done better?” This pre-analysis of the videos allows selecting the passages for discussion. Four coaching appointments are structured according to the learning pyramid. Two medical conversations recorded after the workshop are used as material for coaching.

##### Coaching sessions of the intervention group (four times), one to one

During the first coaching appointment the selected video sequences are watched together with the participating physician. All beneficial communicative techniques used by the physician are named and emphasized by the trainer as positive reinforcement. Afterwards, both trainer and physician reflect on the sequence with regard to “What was good?”/“What could have been done better?” at which the physician’s self-reflection is prioritized compared to the feedback of the trainer.

Consequently, the physician and the coach elaborate specific improvements. After watching and reflecting on all sequences the coaching ends with a summary.

The second session is structured similarly. Here, the second video is analyzed. The experiences of the physicians made after the first coaching is part of the discussion.

The third session is built on a behavior analysis [26]. This method highlights the relation of an incident and the human reaction to it and allows for a critical examination of automatic behavior on four levels (cognitions, emotions, physical reaction and behavior). Thus, key points of the consultations are identified by the coach, focusing on difficult or emotional burdened situations. Patient’s incidents are observed thoroughly by watching the video sequence more than once. Then, physicians’ reactions are analyzed and reflected on the four levels, i.e. cognitions (what came up into your mind?), emotions (what did you feel?), physical reaction (how did you feel?) and behavior (observed verbal and non-verbal reaction). Then, this discussion is reflected and alternative behavior will be developed.

The fourth and last coaching session consists of a case supervision without video. The physician is asked to outline typical difficult conversations with patients and to describe concrete examples. The difficulties are examined according to the four levels as during the third coaching session. Solutions are identified and the results are summarized.

##### Coaching session of the control group (once)

The one coaching session of the control group is structured in line with the first coaching session of the intervention group.

The intervention as well as the control group concludes the coaching by filling out an evaluation questionnaire.

### Outcome: instruments

Based on a reflection of Uitterhoeve and colleagues [27] we aim to adapt our assessment instruments to the teaching content of our training. This approach was used successfully in our previous studies [22, 23] for a checklist as well as for questionnaires. We will evaluate on three different levels: observable behavior by using a checklist and physicians’ as well as patients’ level by using questionnaires.

### Checklist

A checklist (ComOn-Coaching-Checklist) was developed based on the ComOn-Checklist [28]. The ComOn-Coaching-Checklist consists of 13 items covering the beginning of consultation, ending of consultation, structuring consultation, addressing emotions and general communication skills items and can be used to rate oncological consultations (Table 1). Each item is answered by a 5-point rating scale, the endpoints and middle points of which are defined. Raters will be trained in the use of the checklist until an acceptable inter-class correlation will be achieved. This analysis of the videos will provide data to demonstrate whether communication skills will improve after the workshops, as compared to before. Analyses will also compare changes between the intervention and control groups. We will also conduct a second, more personalized evaluation, where the items corresponding to the individual learning goals will be checked. These learning goals are defined in the workshop (*second module*) and will be transferred to the checklist. Those checklist items will be the basis for subsequent practicing in the communication skills training and the coaching. The checklist will be validated in the course of the study and will also serves as a basis for the development of the following questionnaires.

**Table 1:** Checklist ComOn-Coaching

Subjective global evaluation	
How do you assess the communicative competence of the physician in this conversation?	
A	Start of the conversation
A1	Does the physician initiate the conversation appropriately?
A2	Does the physician manage to get an idea of the patient’s perspective at the beginning of, or during the consultation?
B	Structure of conversation
B1	Does the physician actively give structure to the conversation (set an agenda of central topics)?
B2	Does the physician set sub-sections in the course of conversation (in detail)?
C	Emotional issues
C1	Does the physician recognize the patient’s emotions and does he do they name them; evaluation based on NURSE by Back (2008)
C2	Does the physician offer emotional support?
D	End of conversation
D1	Does the physician summarize the content of the consultation and do they close the conversation appropriately?
E	General communication skills
E1	Does the physician use clear and appropriate words during the conversation?
E2	Does the physician use appropriate non-verbal communication during the consultation?
E3	Does the physician adjust his pace during the consultation and does he make appropriate pauses?
E4	Does the physician offer the patient the chance to ask questions during the consultation?
E5	Does the physician check whether the patient has understood the consultation?
F	Overall Evaluation
F1	How do you assess the communication skills of the physician in this conversation?

For the physicians three questionnaires are developed

1. a communication competence self-evaluation questionnaire (Additional file 1) consisting of 16 items responded to using a visual analogue scale of 10 cm: 13 items correspond to those of the checklist, one asks about the general feeling of competence, one about the sense of security when conducting conversations, and one about the communicative knowledge;
2. one questionnaire on expectations for the consultation (Additional file 2) consisting of three questions: two open questions about (a) which topics are important for the physician in the consultation and (b) which communication skills they want to focus on in this consultation, and a third question about their present distress. These are answered by means of a visual-analogue scale; and
3. a third questionnaire on the self-evaluation of the consultation (Additional file 3) consisting of 14 items responded by visual analogue scale: 13 items corresponding to those of the checklist and one refers to the distress experienced during the conversation.

Furthermore the self-evaluation of the consultation before and after the workshop and after the coaching sessions will be compared with special attention to the individual learning goals.

For the patients two questionnaires are developed

1. One questionnaire on expectations for the consultation (Additional file 4) consisting of three questions: two open questions about (a) which topics are important for the patient in the consultation and (b) what they expect from the physician in the communication, and a third question about their present distress. These are answered by means of a visual-analogue scale;
2. a second questionnaire on the evaluation of the consultation (Additional file 5) also consisting of 14 items responded with the help of a visual analogue scale: 13 items correspond to those of the checklist and one refers to the experienced distress during the consultation.

Both physicians and patients will fill out a form on socio-demographic data: physicians additionally will be asked about their specialty and their experience with CSTs; (Additional file 6) patients are asked about their health state (Additional file 7).

Lastly, physicians will evaluate the workshop and the coaching in two separate questionnaires. The main focus is whether the workshop, the coaching and each of their modules have been experienced as well-structured and helpful for their oncological work.

Special attention will be given to the physicians’ learning goals and to fulfillment of the patient expectations by the physician.

### Assessment of outcome measures

At baseline (t0) each physician will fill out the questionnaire on socio-demographic data and the self-evaluation questionnaire about his/her communication competence. Then, two oncological physician-patient consultations will be video recorded with patients who have given their written informed consent. Before each consultation both physician and patient will fill out the questionnaire about their expectations of the consultation and their distress. After the consultation both of them will answer the evaluation questionnaire. After the workshop (t1) the physicians will fill in another self-evaluation questionnaire and record two more consultations. Then the randomization and coaching sessions (once or four times) will take place. After the coaching (t2), another self-evaluation questionnaire will be filled out and two more consultations will be filmed. Three months after the last coaching (catamnesis) the physicians will fill out a last self-evaluation questionnaire (see Fig. 1).

### Sample calculation

For power estimation we refer to the effect sizes of the project “From oncological curative to palliative treatment” [22], the content of which is close to the follow-up project. In this project, depending on the respective communicative skill, medium (ES = .61) to high effects (ES = .78) with an average effect size of ES = .70 were achieved. If the actual effect size of the primary outcome ‘communication skills’ lies at 0.7, with a sample size of 32 per group, a significant difference between the groups with a chance (power) of 80 % can be detected. The calculation was performed using a 2-sided t-test (significance level 5 %). Considering a drop-out rate of 5 % N = 72 physicians in total should be recruited. Then, 6 workshops with 12 participants and 3 trainers will be conducted.

### Randomization

After informed consent of physicians and baseline assessment, participants will be randomized into an intervention and control group by an independent statistician. Results of randomization will be disclosed to the physicians and workshop facilitators after the workshop in order to organize the coaching sessions to avoid biasing the training expectations.

### Statistical analysis

Statistical analysis will be done by independent statisticians. The primary and secondary hypothesis regarding the potential training effects in the transmission of key information and in the improvement of communication skills will be analyzed with a linear mixed model controlling for physicians’ characteristics, study site and multiple measurements. Drop outs will be handled by intention to treat analysis.

### Ethical issues

The study was fully approved by the ethics committees of the University Medical Center Freiburg, Freiburg, Germany, and of the University Hospital *Klinikum rechts der Isar*, Munich, Germany, and is registered under DRKS00004385 in the DRKS (German Clinical Trials Register).

## Discussion

This CST integrates key recommendations of experts in communication skills trainings regarding time, didactics, set up and training [10]. It considers the idea of focusing on workshop-practice transfer by offering intensive coaching sessions after the workshop. A current development in research about CST is the improvement of effect sizes and optimization of didactics. This issue is addressed in this study by comparing the dosage of coaching, i.e. four times in the interventions group and one coaching session for the control group.

There is sufficient empirical data that communication skills can be taught with a special training. However, most studies are evaluated in standardized settings with the help of actor patients, corresponding to level two of Kirkpatrick’s Triangle [7, 29, 30]. According to this model CST can be evaluated on 4 different levels increasing evidence of their actual impact: Physicians’ self-evaluations, knowledge (assessed in standardized consultations), communication skills (assessed in clinic consultations) and patient outcomes. Few studies can provide data on effects of CST on the patient level. The aim of this study is to produce effects on communication skills in real life consultations (level 3) and also to integrate an evaluation of the patients’ views on communication skills. These data help to establish a solid background for assessing patient outcomes in the future.

With this study we also aim to provide evidence allowing generalizing results for physicians. We will recruit physicians working with inpatients as well as with outpatients in different fields of oncology.

The workshop as well as the coaching is individualized, so that physicians can work on their individual learning goals. We expect to successfully train physicians, regardless of their previous experience. This approach allows also concentrated work on specifically identified skills, wherein the physicians are more motivated to work, while the coaching offers time to diagnose additional areas and build motivation for the further work.

Limitations in our results both on the physician and the patient level have to be addressed. As the training is not mandatory it might be that more motivated physicians and those more interested in communication issues will participate. On the patient level, bias may be expected as the patients fill out the questionnaires in the hospital/ambulatory immediately after the consultation and may feel dependent on or committed to their physicians. Furthermore as the consultations are not recorded regularly but particularly for the training the physicians may choose less distressed or “easier” patients as these are more likely to accept the recording. This would limit the generalization to more critical situation and reinforce the ceiling effect of the patient evaluation. This effect is described in literature [31–33] and yet we expect that bias effects may be reduced when physicians see different patients at each assessment point as variability is reduced. Another challenge for our study is the great heterogeneity of the data expected, since different patients, settings, topics and disease stages will be studied. However, this approach strengthens the external validity of our study design.

Assessment on patient level as well as the evaluation of a CST in everyday clinical practice is a challenge. Despite the mentioned limitations we expect to deliver important results for the further development of physician-patient communication.

## Additional files

Additional file 1:
**ComOn Coaching Physician Socio-demographic Data.**

Additional file 2:
**ComOn Coaching Physician Communication Competence Self-Evaluation.**

Additional file 3:
**ComOn Coaching Physician Questionnaire on Expectations for the Consultation.**

Additional file 4:
**ComOn Coaching Physician Questionnaire on the Self-Evaluation of the Consultation.**

Additional file 5:
**ComOn Coaching Patient Socio-demographic and medical Data.**

Additional file 6:
**ComOn Coaching Patient Questionnaire on Expectations for the Consultation.**

Additional file 7:
**ComOn Coaching Patient Questionnaire on the Evaluation of the Consultation.**

## Abbreviations

CST: Communication skills training
ES: Effect size
HCP: Heath care professional
PPC: Physician-patient communication
