# Erratum to: ComOn Coaching: study protocol of a randomized controlled trial to assess the effect of a varied number of coaching sessions on transfer into clinical practice following communication skills training

**DOI:** 10.1186/s12885-015-1602-5

**Published:** 2015-08-25

**Authors:** Marcelo Niglio de Figueiredo, Bärbel Rudolph, Carma L. Bylund, Tanja Goelz, Pia Heußner, Heribert Sattel, Kurt Fritzsche, Alexander Wuensch

**Affiliations:** 1 Department of Psychosomatic Medicine and Psychotherapy, Freiburg University Medical Center, Hauptstr. 8, Freiburg, Germany; 2 Clinic of Dermatology and Venereology, Freiburg University Medical Center, Hauptstr. 7, Freiburg, Germany; 3 Department of Psychosomatic Medicine and Psychotherapy, Klinikum rechts der Isar, Technical University Munich, Langerstr. 3, Munich, Germany; 4 Department of Medical Education, Hamad Medical Corporation, Doha, Qatar; 5 Weill-Cornell Medical College, Doha, Qatar; 6 Center for Pediatrics, Freiburg University Medical Center, Mathildenstr. 6, Freiburg, Germany; 7 Department of Haematology and Internal Oncology, Interdisciplinary Psycho-Oncology Center, University Clinic of Munich – Grosshadern, Marchioninistr. 15, Munich, Germany

Unfortunately, the original version of this article [1] contained an error in the author list. The name of the author Bärbel Rudolph was incorrectly spelt as Bärbel Rodolph. The correct spelling is Bärbel Rudolph.
