# Supplementary material for: ComOn Coaching: Study protocol of a randomized controlled trial to assess the effect of a varied number of coaching sessions on transfer into clinical practice following communication skills training
Source: BMC Cancer. 2015 Jul 7;15:503. doi: 10.1186/s12885-015-1454-z (PMC4494160; doi:10.1186/s12885-015-1454-z)
Supplement: Additional file 1: — ComOn Coaching Physician Socio-demographic Data. [file 12885_2015_1454_MOESM1_ESM.docx]

# Freiburg Medical Center

***COM-ON***

*communication skills in oncology*

**Psychosomatic Medicine and Psychotherapy**

Director: Prof. Dr. Michael Wirsching

**in Cooperation with the CCCF**, Director: Prof. J. Duyster

Klinikum rechts der Isar, TU München

**Kommunikative Kompetenzen in der Onkologie**

*Freiburger Trainingsprogramm*

**Psychosomatic Medicine and Psychotherapy**

Director: Prof. Dr. Peter Henningsen

**in Cooperation with the RHCCC**,

Director: Prof. P. Herschbach

**Contact in Freiburg**

Marcelo de Figueiredo, Dipl.-Psychologist

Tel.: +49 761 / 270 68809

E-Mail: marcelo.de.figueiredo@uniklinik-freiburg.de

Johanna Freund, Dipl.-Psychologist

Tel.: +49 761 / 270 68809

E-Mail: johanna.freund@uniklinik-freiburg.de

**Contact in Munich**

Dr. Alexander Wünsch, Dipl.-Psychologe

Tel.: +49 89 / 4140 4316

E-Mail: a.wuensch@tum.de

**ComOn Coaching: Communication in oncology**

**Assessment of the communicative competence**

Self-evaluation by the **physician**

Dear participant,

on the following page you are asked to answer some questions about your experience with oncological consultations.

Please answer the questions thoroughly.

The data will be treated with the utmost discretion, analyzed according to the laws of information privacy and used for scientific purposes only.

**Please turn over →**

**Physician code**: |_||_||_||_| |_||_|

Day and month of your birthday Initial letters of the name of your mother

Date |_||_||_||_||_||_| Time |_||_|:|_||_|

Assessment: t |_|

**The following example shows you how to answer the questions:**

Each affirmation is followed by a 10 cm long line. This line is placed between two poles, e.g. “very secure” und “very unsecure”. You answer the question making a stroke in the line: the closer to “very secure” you make the stroke, the more secure you are; the closer to “very unsecure” you make the stroke, the more unsecure you are. There is no right or wrong: important is your personal opinion.

If you need to correct your answer (e.g. because you made the stroke on the wrong place), please cancel clearly the “wrong” stroke – as in the following example – and make a new one on the correct spot.

In the example the physician thought, after he made his stroke, he actually feels more secure about, how to begin a consultation than he crossed. He then canceled the first stroke and made a new one closer to “very secure”.

**Please turn over →**

**How do you evaluate the consultations you conduct with oncological patients?**

*I feel...*

_A1_ very secure very unsecure

*About how to* ***iniciate*** *the consultation.*

*I find it quite…*

_A2_ easy difficult

*to get an idea of* ***the patient’s perspective****.*

*I find it quite…*

_B1_ easy difficult

*to* ***structure*** *the consultation* *and* ***set an agenda*** *of central topics.*

*I find it quite…*

_B2_ easy difficult

*to* ***organize the themes*** *in sub-sections.*

*I find it quite…*

_C1_ easy difficult

*to recognize the* ***patient’s emotions****and to* ***name*** *them.*

*I find it quite…*

_C2_ easy difficult

*to offer the patient* ***emotional support.***

*I find it quite…*

_E1_ easy difficult

*to use* ***clear and appropriate words****.*

*I find it quite…*

_E2_ easy difficult

*to use appropriate* ***non-verbal communication.***

**Please turn over →**

*I find it quite…*

_E3_ easy difficult

*to use* ***adjust my (speech) pace*** *during the consultation and to make appropriate* ***pauses****.*

*I find it quite…*

_E4_ easy difficult

*to give the patient the chance to ask* ***questions.***

*I find it quite…*

_E5_ easy difficult

*to check whether the* ***patient has understood*** *the consultation.*

*I find it quite…*

_D1_ easy difficult

*to* ***summarize*** *the content of the conversation and to* ***close it*** *appropriately.*

*The* ***overall quality*** *of the oncological consultations I conduct is…*

_F1_ very good very bad.

*In oncological consultations* ***I feel****...*

_F2_ very secure very unsecure

*I have ...*

_G1_ very good very poor

***theoretical knowledge*** *about conducting oncological consultations state-of-the-art.*

*I can transfer my theoretical* ***knowledge****...*

_G2_ very well very poorly

*in the everyday praxis.*

**Please turn over →**

*Oncological consultations are...*

_X_ not at all very.

**emotionally distressing** for me.

**(Following questions were made only in t0)**

*Are there other communicative situations with persons or groups that you find difficult?*

__________________________________________________________________________

__________________________________________________________________________

__________________________________________________________________________

__________________________________________________________________________

*Are there other topics that are difficult for you in communication?*

__________________________________________________________________________

__________________________________________________________________________

__________________________________________________________________________

__________________________________________________________________________

**(Following question was made in t1 and t2)**

*In the workshop you formulated your individual learning goals. In relation to your first goal, how would you rate your present state?*

_y_  worse -2 -1 0 1 2 3 4 goal completely achieved.

**Thank you for the information!**
