# Supplementary material for: ComOn Coaching: Study protocol of a randomized controlled trial to assess the effect of a varied number of coaching sessions on transfer into clinical practice following communication skills training
Source: BMC Cancer. 2015 Jul 7;15:503. doi: 10.1186/s12885-015-1454-z (PMC4494160; doi:10.1186/s12885-015-1454-z)
Supplement: Additional file 2: — ComOn Coaching Physician Communication Competence Self-Evaluation. [file 12885_2015_1454_MOESM2_ESM.docx]

# Freiburg Medical Center

***COM-ON***

*communication skills in oncology*

**Psychosomatic Medicine and Psychotherapy**

Director: Prof. Dr. Michael Wirsching

**in Cooperation with the CCCF**, Director: Prof. J. Duyster

Klinikum rechts der Isar, TU München

**Kommunikative Kompetenzen in der Onkologie**

*Freiburger Trainingsprogramm*

**Psychosomatic Medicine and Psychotherapy**

Director: Prof. Dr. Peter Henningsen

**in Cooperation with the RHCCC**,

Director: Prof. P. Herschbach

**Contact in Freiburg**

Marcelo de Figueiredo, Dipl.-Psychologist

Tel.: +49 761 / 270 68809

E-Mail: [marcelo.de.figueiredo@uniklinik-freiburg.de](mailto:marcelo.de.figueiredo@uniklinik-freiburg.de)

Johanna Freund, Dipl.-Psychologist

Tel.: +49 761 / 270 68809

E-Mail: johanna.freund@uniklinik-freiburg.de

**Contact in Munich**

Dr. Alexander Wünsch, Dipl.-Psychologe

Tel.: +49 89 / 4140 4316

E-Mail: a.wuensch@tum.de

**ComOn Coaching: Communication in oncology**

**Assessment of your expectations for the upcoming consultation**

Self-evaluation by the **physician**

Dear participant,

on the following page you are asked to answer some questions about your expectations concerning the upcoming consultation.

Please answer the questions thoroughly.

The data will be treated with the utmost discretion, analyzed according to the laws of information privacy and used for scientific purposes only.

**Please turn over →**

**Physician code**: |_||_||_||_| |_||_|

Day and month of your birthday Initial letters of the name of your mother

Date |_||_||_||_||_||_| Time |_||_|:|_||_|

Assessment: t |_|

1. **Which main topic is important for you in the following consultation?**

___________________________________________________________________

___________________________________________________________________

___________________________________________________________________

___________________________________________________________________

___________________________________________________________________

1. **On what aspects of communication do you want to pay special attention to in this consultation?**

___________________________________________________________________

___________________________________________________________________

___________________________________________________________________

___________________________________________________________________

___________________________________________________________________

1. **How emotionally distressed are you concerning the following consultation?**

*You answer this question making a stroke in the visual-analog-scale: the closer to „not distressed“ you make the stroke, the less distressed you are; the closer to “very distressed” you make the stroke, the more distressed you are*

Not distressed very distressed

**Thank you for your opinion!**
