# Supplementary material for: ComOn Coaching: Study protocol of a randomized controlled trial to assess the effect of a varied number of coaching sessions on transfer into clinical practice following communication skills training
Source: BMC Cancer. 2015 Jul 7;15:503. doi: 10.1186/s12885-015-1454-z (PMC4494160; doi:10.1186/s12885-015-1454-z)
Supplement: Additional file 3: — ComOn Coaching Physician Questionnaire on Expectations for the Consultation. [file 12885_2015_1454_MOESM3_ESM.docx]

# Freiburg Medical Center

***COM-ON***

*communication skills in oncology*

**Psychosomatic Medicine and Psychotherapy**

Director: Prof. Dr. Michael Wirsching

**in Cooperation with the CCCF**, Director: Prof. J. Duyster

Klinikum rechts der Isar, TU München

**Kommunikative Kompetenzen in der Onkologie**

*Freiburger Trainingsprogramm*

**Psychosomatic Medicine and Psychotherapy**

Director: Prof. Dr. Peter Henningsen

**in Cooperation with the RHCCC**,

Director: Prof. P. Herschbach

**Contact in Freiburg**

Marcelo de Figueiredo, Dipl.-Psychologist

Tel.: +49 761 / 270 68809

E-Mail: marcelo.de.figueiredo@uniklinik-freiburg.de

Johanna Freund, Dipl.-Psychologist

Tel.: +49 761 / 270 68809

E-Mail: johanna.freund@uniklinik-freiburg.de

**Contact in Munich**

Dr. Alexander Wünsch, Dipl.-Psychologe

Tel.: +49 89 / 4140 4316

E-Mail: a.wuensch@tum.de

**ComOn Coaching: Communication in oncology**

**Self-evaluation of the consultation**

Questionnaire for the **physician**

Dear participant,

on the following page you are asked to answer some questions about **the consultation you just conducted**.

Please answer the questions thoroughly.

The data will be treated with the utmost discretion, analyzed according to the laws of information privacy and used for scientific purposes only.

**Please turn over →**

**Physician code**: |_||_||_||_| |_||_|

Day and month of your birthday Initial letters of the name of your mother

Date |_||_||_||_||_||_| Time |_||_|:|_||_|

Assessment: t |_|

**The following example shows you how to answer the questions:**

Each affirmation is followed by a 10 cm long line. This line is placed between two poles, e.g. “satisfied” and “unsatisfied”. You answer the question making a stroke in the line: the closer to “satisfied“ you make the stroke, the more satisfied you are; the closer to “unsatisfied” you make the stroke, the more unsatisfied you are. There is no right or wrong: important is your personal opinion.

If you need to correct your answer (e.g. because you made the stroke on the wrong place), please cancel clearly the “wrong” stroke – as in the following example – and make a new one on the correct spot.

In the example the physician thought, after he made his stroke, that he was more satisfied with the way he began the consultation than he crossed. He then canceled the first stroke and made a new one closer to “satisfied”.

**Please turn over →**

**How did you perceive the consultation you just conducted?**

*I was...*

_A1_ satisfied unsatisfied

*with the way I* ***initiated*** *the conversation.*

*In this consultation I got an idea of* ***the patient’s perspective****.*

_A2_ agree disagree

*I actively* ***structured*** *the consultation and* ***set an agenda*** *of central topics…*

_B1_ very well poorly.

*I could* ***organize the sub-sections*** *in the course of the conversation…*

_B2_ very well poorly.

*I was able to recognize the* ***patient’s emotions*** *and* ***name them****…*

_C1_ very well poorly.

*I was able to offer the patient* ***emotional support****…*

_C2_ agree disagree

*I used throughout* ***clear and appropriate words****…*

_E1_ agree disagree

*I used appropriate* ***non-verbal communication****…*

_E2_ agree disagree

*I could* ***adjust my pace*** *when talking and made appropriate* ***pauses****…*

_E3_ agree disagree

*I offered the patient the chance to ask* ***questions****.*

_E4_ agree disagree

*I checked whether* ***the patient had understood*** *the consultation.*

_E5_ agree disagree

**Please turn over →**

*I was...*

_D1_ satisfied unsatisfied

*with the way I* ***summarized*** *the content of consultation* *and* ***closed it****.*

*The* ***overall quality*** *of this consultation was…*

_F1_ very good very bad.

*This consultation* ***distressed*** *me* ***emotionally****...*

_X_ not at all very much.

*Before the consultation you were asked about the* ***topics*** *that were important for you. How did you achieve them?*

very well poorly

*Before the consultation you were asked about the* ***communication skills*** *you wanted to focus on. How did you achieve this?*

very well poorly

**Thank you for your opinion!**
