# Supplementary material for: ComOn Coaching: Study protocol of a randomized controlled trial to assess the effect of a varied number of coaching sessions on transfer into clinical practice following communication skills training
Source: BMC Cancer. 2015 Jul 7;15:503. doi: 10.1186/s12885-015-1454-z (PMC4494160; doi:10.1186/s12885-015-1454-z)
Supplement: Additional file 5: — ComOn Coaching Patient Socio-demographic and medical Data. [file 12885_2015_1454_MOESM5_ESM.docx]

# Freiburg Medical Center

***COM-ON***

*communication skills in oncology*

**Psychosomatic Medicine and Psychotherapy**

Director: Prof. Dr. Michael Wirsching

**in Cooperation with the CCCF**, Director: Prof. J. Duyster

Klinikum rechts der Isar, TU München

**Kommunikative Kompetenzen in der Onkologie**

*Freiburger Trainingsprogramm*

**Psychosomatic Medicine and Psychotherapy**

Director: Prof. Dr. Peter Henningsen

**in Cooperation with the RHCCC**,

Director: Prof. P. Herschbach

**Contact in Freiburg**

Marcelo de Figueiredo, Dipl.-Psychologist

Tel.: +49 761 / 270 68809

E-Mail: marcelo.de.figueiredo@uniklinik-freiburg.de

Johanna Freund, Dipl.-Psychologist

Tel.: +49 761 / 270 68809

E-Mail: johanna.freund@uniklinik-freiburg.de

**Contact in Munich**

Dr. Alexander Wünsch, Dipl.-Psychologe

Tel.: +49 89 / 4140 4316

E-Mail: a.wuensch@tum.de

**ComOn Coaching: Communication in oncology**

**Evaluation of the consultation**

Questionnaire for the **patient**

Dear patient,

in the following page you are asked to answer some questions about **the consultation you just had**. We would like to know, how the conversation was *for you*. There is no right or wrong answers: we are interested in your sincere, personal opinion.

Please answer the questions thoroughly.

The data will be treated with the utmost discretion, analyzed according to the laws of information privacy and used for scientific purposes only.

**Please turn over →**

**Physician code**: |_||_||_||_| |_||_|

Day and month of your birthday Initial letters of the name of your mother

Date |_||_||_||_||_||_| Time |_||_|:|_||_|

Assessment: t |_|

**The following example shows you how to answer the questions:**

Each affirmation is followed by a 10 cm long line. This line is placed between two poles, e.g. “satisfied” and “unsatisfied”. You answer the question making a stroke in the line: the closer to “satisfied” you make the stroke, the more satisfied you are; the closer to “unsatisfied” you make the stroke, the more unsatisfied you are. There is no right or wrong: important is your personal opinion.

If you need to correct your answer (e.g. because you made the stroke on the wrong place), please cancel clearly the “wrong” stroke – as in the following example – and make a new one on the correct spot.

In the example the patient thought, after he made his stroke, that the physician initiated the consultation better than he crossed. She then canceled first stroke and made a new one closer to “satisfied”.

**Please turn over →**

**How was the consultation for you?**

*I was...*

_A1_ satisfied unsatisfied

*with the way the physician* ***initiated*** *the conversation.*

*During this consultation the physician got an idea of* ***how I understand my situation****.*

_A2_ agree disagree

*The physician* ***structured*** *the consultation* *and* ***set an agenda*** *of central topics …*

_B1_ very well poorly.

*The physician could* ***organize the subsections*** *in the course of the conversation…*

_B2_ very well poorly.

*The physician was able to recognize* ***my emotions.***

_C1_ very well poorly.

*The physician was able to offer me* ***emotional support.***

_C2_ agree disagree

*The physician used throughout* ***clear and appropriate words****.*

_E1_ agree disagree

*The physician used appropriate* ***non-verbal communication****.*

_E2_ agree disagree

*The physician* ***adjusted his pace*** *when talking and made appropriate* ***pauses****.*

_E3_ agree disagree

*The physician offered me the chance to ask* ***questions****.*

_E4_ agree disagree

*The physician checked wheter* ***I understood*** *the consultation.*

_E5_ agree disagree

**Please turn over →**

*I was...*

_D1_ satisfied unsatisfied

*with the way the physician* ***summarized*** *the consultation* *and* ***closed it****.*

*The* ***overall quality*** *of this consultation was*

_F1_ very good very bad

*This consultation* ***distressed*** *me* ***emotionally****...*

_X_ not at all very much.

*Before the consultation you were asked about the* ***topics*** *that were important for you. How were they discussed?*

very good poorly

*Before the consultation you were asked about* ***your expectations about the way the physician communicates with you.*** *How did she/he achieve this?*

very good poorly

**Thank you for your opinion!**
