# Supplementary material for: ComOn Coaching: Study protocol of a randomized controlled trial to assess the effect of a varied number of coaching sessions on transfer into clinical practice following communication skills training
Source: BMC Cancer. 2015 Jul 7;15:503. doi: 10.1186/s12885-015-1454-z (PMC4494160; doi:10.1186/s12885-015-1454-z)
Supplement: Additional file 6: — ComOn Coaching Patient Questionnaire on Expectations for the Consultation. [file 12885_2015_1454_MOESM6_ESM.docx]

# Freiburg Medical Center

***COM-ON***

*communication skills in oncology*

**Psychosomatic Medicine and Psychotherapy**

Director: Prof. Dr. Michael Wirsching

**in Cooperation with the CCCF**, Director: Prof. J. Duyster

Klinikum rechts der Isar, TU München

**Kommunikative Kompetenzen in der Onkologie**

*Freiburger Trainingsprogramm*

**Psychosomatic Medicine and Psychotherapy**

Director: Prof. Dr. Peter Henningsen

**in Cooperation with the RHCCC**,

Director: Prof. P. Herschbach

**Contact in Freiburg**

Marcelo de Figueiredo, Dipl.-Psychologist

Tel.: +49 761 / 270 68809

E-Mail: marcelo.de.figueiredo@uniklinik-freiburg.de

Johanna Freund, Dipl.-Psychologist

Tel.: +49 761 / 270 68809

E-Mail: johanna.freund@uniklinik-freiburg.de

**Contact in Munich**

Dr. Alexander Wünsch, Dipl.-Psychologe

Tel.: +49 89 / 4140 4316

E-Mail: a.wuensch@tum.de

**ComOn Coaching: Communication in oncology**

**Socio-demographic Data**

**Physician**

Dear participant,

on the following page you are asked to give some socio-demographic information about you.

Please answer the questions thoroughly.

The data will be treated with the utmost discretion, analyzed according to the laws of information privacy and used for scientific purposes only.

**Please turn over →**

**Physician code**: |_||_||_||_| |_||_|

Day and month of your birthday Initial letters of the name of your mother

Date |_||_||_||_||_||_| Time |_||_|:|_||_|

**Socio-demographic Information**

Sex: male |_|_1_ female |_|_2_

Age: |_||_| Years

Resident: no |_|_0_ yes |_|_1_ Year (1-5): |_|

Medical specialist: no |_|_0_ yes |_|_1_

Area of Specialization: _________________________________

: |_||_| Year

In oncology: |_||_| Year

Estimated percentage of oncological patients last trimester: |_||_||_| %

Previous participation in courses on basics of psychosomatic medicine:

Number of hours: |_||_||_|

Name of the course(s): _______________________________________________________

Previous participation on communication-workshops:

Number of hours: |_||_||_|

Name of the course(s): _______________________________________________________

__________________________________________________________________________

**Thank you for the information!**
