# Supplementary material for: ComOn Coaching: Study protocol of a randomized controlled trial to assess the effect of a varied number of coaching sessions on transfer into clinical practice following communication skills training
Source: BMC Cancer. 2015 Jul 7;15:503. doi: 10.1186/s12885-015-1454-z (PMC4494160; doi:10.1186/s12885-015-1454-z)
Supplement: Additional file 7: — ComOn Coaching Patient Questionnaire on the Evaluation of the Consultation. [file 12885_2015_1454_MOESM7_ESM.docx]

# Freiburg Medical Center

***COM-ON***

*communication skills in oncology*

**Psychosomatic Medicine and Psychotherapy**

Director: Prof. Dr. Michael Wirsching

**in Cooperation with the CCCF**, Director: Prof. J. Duyster

Klinikum rechts der Isar, TU München

**Kommunikative Kompetenzen in der Onkologie**

*Freiburger Trainingsprogramm*

**Psychosomatic Medicine and Psychotherapy**

Director: Prof. Dr. Peter Henningsen

**in Cooperation with the RHCCC**,

Director: Prof. P. Herschbach

**Contact in Freiburg**

Marcelo de Figueiredo, Dipl.-Psychologist

Tel.: +49 761 / 270 68809

E-Mail: marcelo.de.figueiredo@uniklinik-freiburg.de

Johanna Freund, Dipl.-Psychologist

Tel.: +49 761 / 270 68809

E-Mail: johanna.freund@uniklinik-freiburg.de

**Contact in Munich**

Dr. Alexander Wünsch, Dipl.-Psychologe

Tel.: +49 89 / 4140 4316

E-Mail: a.wuensch@tum.de

**ComOn Coaching: Communication in oncology**

**Socio-demographic data**

**Patient**

Code |_||_||_||_||_||_| t|_|

Date |_||_||_||_||_||_| Time |_||_|:|_||_|

**Personal information**

(Questions on the patient)

Sex: male |_|_1_ female |_|_2_

Age: |_||_| Years

Citzenship: |_|_1_ German |_|_2_ Other: _______________________

Do you live in a stable partnership? |_|_1_ yes |_|_2_ no

Do you have children? |_|_1_ yes |_|_2_ no

How do you live presently?

|_|_1_ alone |_|_2_ with partner |_|_3_ with partner and children

|_|_4_ alone with children |_|_5_ at your parent’s |_|_6_ other: _______________

Which is your present occupational status?

|_|_1_ employed / in formation

|_|_2_ certified unfit for work

|_|_3_ non-employed / in pension

Which is your higher educational achievement?

|_|_1_ secondary school (9^th^ grade)

|_|_2_ middle school (10^th^ grade)

|_|_3_ Baccalaureate (12^th^ grade)

|_|_4_ University

**Please turn over →**

**Medical Information**

(Questions on the physician)

Diagnosis

______________________________________________

|_| not known, unclear

Date of the initial diagnosis (Month/Year): _______/____

Disease status?

|_|_1_ first tumor |_|_2_ second tumor |_|_3_ relapse

|_|_4_ remission |_|_5_ unclear

Were metastasis diagnosed? |_|_1_ yes |_|_2_ no |_|_3_ unknown

Which treatment did you get in the last two months? (Multiple selections possible)

|_|_1_ surgery |_|_2_ chemotherapy |_|_3_ radiotherapy

|_|_4_ hormone therapy |_|_5_ other: ____________________ |_|_6_ none

Which treatment is planned? (Multiple selections posible)

|_|_1_ surgery |_|_2_ chemotherapy |_|_3_ radiotherapy

|_|_4_ hormone therapy |_|_5_ other: ____________________ |_|_6_ none

Which is the treatment status of the patient?

|_|_1_ palliative |_|_2_ curative |_|_3_ unclear

Has the patient other important diseases?

|_|_1_ yes, following: ______________________

|_|_2_ no
